# A systematic review of resilience and mental health outcomes of conflict-driven adult forced migrants

**DOI:** 10.1186/1752-1505-8-13

**Published:** 2014-08-20

**Authors:** Chesmal Siriwardhana, Shirwa Sheik Ali, Bayard Roberts, Robert Stewart

**Affiliations:** 1 Department of Psychological Medicine, Institute of Psychiatry, King’s College London, PO Box 92, De Crespigny Park, London SE5 8AF, UK; 2 King’s College London School of Medicine, London, UK; 3 London School of Hygiene and Tropical Medicine, London, UK

**Keywords:** Mental health, Forced migration, Adult resilience

## Abstract

**Background:**

The rising global burden of forced migration due to armed conflict is increasingly recognised as an important issue in global health. Forced migrants are at a greater risk of developing mental disorders. However, resilience, defined as the ability of a person to successfully adapt to or recover from stressful and traumatic experiences, has been highlighted as a key potential protective factor. This study aimed to review systematically the global literature on the impact of resilience on the mental health of adult conflict-driven forced migrants.

**Methodology:**

Both quantitative and qualitative studies that reported resilience and mental health outcomes among forcibly displaced persons (aged 18+) by way of exploring associations, links, pathways and causative mechanisms were included. Fourteen bibliographic databases and seven humanitarian study databases/websites were searched and a four stage screening process was followed.

**Results:**

Twenty three studies were included in the final review. Ten qualitative studies identified highlighted family and community cohesion, family and community support, individual personal qualities, collective identity, supportive primary relationships and religion. Thirteen quantitative studies were identified, but only two attempted to link resilience with mental disorders, and three used a specific resilience measure. Over-reliance on cross-sectional designs was noted. Resilience was generally shown to be associated with better mental health in displaced populations, but the evidence on this and underlying mechanisms was limited.

**Discussion:**

The review highlights the need for more epidemiological and qualitative evidence on resilience in forcibly displaced persons as a potential avenue for intervention development, particularly in resource-poor settings.

## Introduction

The rising global burden of forced migration due to armed conflict is increasingly recognised as an important issue in international public health. It is estimated that there are around 45.2 million forced migrants globally [1,2]. Conflict-driven forced migration has been shown to have a strong association with higher levels of mental disorders among affected migrant populations [3,4]. Forced migrants can be divided into two broad categories of externally displaced (across national boundaries) and internally displaced (within national borders), the latter not protected by international refugee laws and therefore prevented from accessing international aid and services [5,6]. However, in most conflict situations, reasons for displacement and subsequent problems faced are strikingly similar for both external and internal migrant groups [6].

The process of displacement can be broadly categorised into pre-flight, flight and post-flight/resettlement phases [7]. These different phases of displacement are associated with particular groups of risk factors for mental ill health of displaced populations [6]. The adverse impact of migration on mental health is well established [3,8], and the multifaceted causation is related with exposure to traumatic events, daily stressors and impoverishment [9]. Compounded by issues associated with pre-existing vulnerabilities, migration episode stress and post-migration environments, trauma exposure may predict the potential impact of forced displacement and subsequent development of psychopathology such as depression, anxiety and PTSD [3,6]. Internally displaced people (IDP) have been shown to be associated with higher levels of psychological morbidity than refugee populations [3,10].

However, it has been established that many of those who experience conflict-driven, often highly-traumatic forced migration do not develop mental disorders despite being at-risk [8]. Factors such as individual and/or community resilience and social support have been highlighted as key potential mediators between forced migration experience and subsequent mental health impact [11]. Individual resilience has been described as the ability of a person to successfully adapt to or recover from stressful and traumatic experiences [12]. Resilience is conceptualised today as a multidimensional construct that incorporates personal skills and qualities together with social environments and a supportive family network, rather than a complex of purely personal attributes such as self-esteem or hardiness [13]. Resilience is seen as a dynamic process that alters according to cultural, developmental and historical context of individuals, varying across age and gender [13]. Similarly, community resilience is seen as the collective ability to adapt and recover from adversity as a population or a community [14-17]. However, similar to the discussion and debate around the concept of individual resilience, the concept of community resilience is also constantly evolving [18,19].

Studies have found that poor levels of resilience among displaced individuals/populations may predict the development of psychopathology [11,20]. Prolonged displacement, continuing adversity and older age have been shown to be associated with decreased resilience, in turn linked to mental ill health [11]. On the other hand, enhanced socio-economic conditions, younger age and social support have been linked to increased resilience and better mental health outcomes, especially in adolescent refugee groups [11,21]. Therefore, the construct of resilience may form an essential element of epidemiological and interventional research aiming to improve mental health outcomes among conflict-driven forced migrants. The Inter Agency Standing Committee (IASC) guidelines on Mental Health and Psychosocial Support in Emergency Settings refer to resilience as an important element for consideration [22].

Despite the evidence that resilience may be a key factor associated with mental health outcomes in forced migration and its potential importance for reducing mental disorder burden among displaced populations, synthesis of the available evidence in the form of systematic reviews are limited. Tol et al., [23] reviewed studies focused on resilience and mental health of children and adolescents affected by conflict in low and middle income countries (LMIC). They concluded that studies were small in number, mainly cross-sectional and had limited focus on the dynamic and complex nature of resilience [23]. Their review did not explore adult conflict-affected populations and excluded studies originating from high income countries (HIC), thereby missing populations such as refugees or asylum seekers. We aimed to fill this gap in evidence by collating the current evidence on resilience and mental health outcomes of conflict-driven adult forced migrants. Our systematic review is reported in accordance with PRISMA guidelines [24].

## Methods

### Inclusion and exclusion criteria

For the purpose of this systematic review, the population of interest were adults aged 18 years and over, affected by conflict, internally or externally displaced and living in any country. Studies conducted among children or adolescents (less than 18 years of age) were excluded, as these had been recently reviewed elsewhere [23]. However, studies involving individuals who were displaced as children due to conflict but are currently adults (e.g. those displaced during WW II as children) were considered. In addition, mixed-population studies (those that include displaced and non-displaced populations within the sample; e.g. IDP and non-IDP residents) were also included. Primary quantitative and qualitative research studies that reported specifically on resilience and its impact on mental health of displaced persons by way of exploring associations, links, pathways and causative mechanisms were included to get a broader insight into how the relationships between resilience and mental health have been explored. However, studies primarily investigating mental health outcomes but not resilience and studies investigating physical health or other migration-specific outcomes, not directly related to mental health, were excluded. Books, book chapters, conference proceedings, reviews, editorials, reports, dissertations and other similar publications were excluded.

### Information sources and data search

Quantitative and qualitative studies from both published and grey literature were identified from thirteen bibliographic databases and seven humanitarian study databases and websites. Bibliographic databases searched included Anthropological Index Online, BIOLINE, BIOMED CENTRAL, Cambridge Scholarly Articles, Cochrane Library, OVID, Psych Info, Pub Med, Science Direct Elsevier, Social Services Abstracts, UN, Web of Knowledge, Médecins Sans Frontières Field Research and Embase. The humanitarian agency websites included Relief Web, Forced Migration Online, Internal Displacement Monitoring Centre, World Health Organisation, United Nations High Commissioner for Refugees, International Committee for the Red Cross and the International Organisation for Migration.

Standard search procedures were followed. Limits were not placed on the start publication date. The end publication date was 31 January 2014. The review was limited to English language publications. The search terms were chosen according to the search requirements of the databases or websites, and reviewed via looking at emerging publications. The search terms were: [Resilien* AND mental health AND internally displaced OR internally displaced people OR IDP OR forced migrants OR refugee* OR asylum seeker*]. Ethical approval was deemed not necessary to be obtained for this review as data are publicly available.

### Study screening and selection

**Figure 1:**
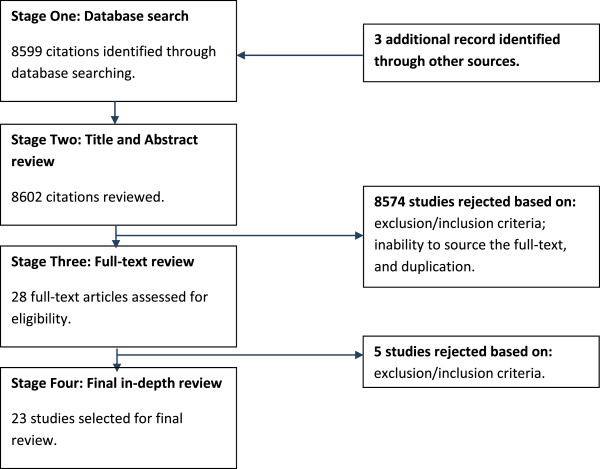
Literature screening process flow diagram.

Literature for the review was selected through a four-step screening process (Figure 1). The databases and websites described above were searched using the defined search terms, and citations were identified (step 1). The title and abstract of the identified citations were screened for inclusion and exclusion criteria (step 2). Subsequently, the full text of selected publications from step 2 was screened for re-confirmation of matching inclusion criteria and methodological issues (step 3). The remaining studies were then subjected to final in-depth review (step 4). At this last stage, quantitative and qualitative studies were reviewed separately using appropriate guidance. A quantitative and qualitative synthesis of the included study findings was conducted based on the study population, measurement of mental health, measurement of resilience, and links between resilience and mental health.

## Results

The results from the selection procedure are detailed in Table 1; 8599 citations were obtained via the initial database search and three additional record identified by manually searching through journals. These included results from the grey literature such as humanitarian websites. A title and abstract review was then conducted as part of stage two, primarily based on the exclusion/inclusion criteria; inability to source the full-text, and duplication (8574 rejected). A further full-text review was conducted as part of stage three, based on the exclusion/inclusion criteria (5 studies rejected). Twenty three studies remained for the final in-depth review: Davis et al., 2000, Almedom et al., 2005, Almedom et al., 2007, Jamil et al., 2007, Schweitzer et al., 2007, Pedersen et al., 2008, Sossou et al., 2008, Kuwert et al., 2009, Beiser et al., 2010, Hooberman et al., 2010, Somasundaram & Sivayokan., 2010, Andersson et al., 2011, Araya et al., 2011, Beiser et al., 2011, Nuwayhid et al., 2011, Thomas et al., 2011, Bhui et al., 2012, Fernando, 2012, Lenette et al., 2013, Chung et al., 2013, Lewis, 2013, Suarez, 2013, Arnetz et al., 2013 (see Table 1) [25-47].

**Table 1:** Overview of included studies (N = 23)

**Study characteristics**	**Qualitative (Total =10)**	**Quantitative (Total = 13)**	
**Region**			
Europe	0	3	
North America/Canada	3	4	
Latin America and the Caribbean	0	2	
Africa	0	4	
Australasia	7	0	
**Population**			
IDPs	2	5	
Refugees/Asylum seekers	7	3	
Mixed (non-displaced/displaced)	1	3	
Mixed (other)	0	2	
**Sample size**			
<100	8	3	
100-500	0	7	
501>	0	3	
Not reported	2	0	
**Sample selection**			
Convenience	10	3	
Random (cross-sectional, cohort etc.)	0	10	
**Displacement period**			
Post-migration	9	13	
Other	1	0	

Studies included in the final in-depth review had publication dates ranging from 2000 to 2013, 12 of which were primarily quantitative studies (Tables 2 and 3) and 1 study was of mixed-method design [25]. The remaining 10 were qualitative studies (Table 4). The majority of quantitative studies were cross-sectional (10 in total), 2 were case–control studies [26,27] and 1 was of cohort design [28]. Two studies used qualitative data gleaned from participants interrogating the primary resilience-measuring study instrument to enrich the discussion sections [29,30]. However, as both of them primarily presented quantitative findings, they were categorised as quantitative studies.

**Table 2:** Overview of quantitative studies

**Study**	**Sample and background**	Resilience outcome measures*	Mental health outcome and other measures*	Validity/reliability of** measures/language	Statistical test**
**Almedom et al.,**[29]	265 Eritrean IDP camp dwellers and urban (non-displaced) residents	SOC-13	Not specified	SOC-13 translated, adapted, used in 9 Eritrean languages	t-test (2 tailed), Analysis of Variance (RR)
**Jamil et al.,**[31]	116 adult Iraqi refugees resettled in the United States	Not specified	HSCL-25, PDS	Translated/used in Arabic. HSCL-25 Cα; 0.95, 0.88, PDS Cα; 0.94	Descriptive statistics, Pearson chi square
**Almedom et al.,**[29]	265 Eritrean IDP camp dwellers and urban (non-displaced) residents	SOC-13	Not specified	SOC-13 translated, adapted, used in 9 Eritrean languages	t-test (2 tailed), Analysis of Variance (RR)
**Pedersen et al.,**[25]	373 adults across five Peruvian rural/semi-rural settlements	Not specified	GHQ-12, HSCL-25, TQ	All instruments subjected to cultural and semantic validation for Quechua language use	Descriptive statistics, Generalized linear regression coefficients
**Kuwert et al.,**[32]	1513 German participants aged >61 (239 displaced in WWII)	RS-11	PHQ-2, GAD-7, FLZ^M^	No information on translation/adaptation. RS-11 Cα;0.91, PHQ-2 Cα;0.78, GAD-7 Cα;0.89, FLZ^M^ Cα;0.83	*X*^*2*^ test, MANOVA, Stepwise linear regression coefficients
**Beiser et al.,**[33]	100 adult residents from Niger Delta region of Nigeria	Information on exposure to political conflict, social capital, perceived social support	WHO-CIDI-K for PTSD	Translated to Ogoni language, further convergent validation.	Descriptive statistics, correlation coefficients, stepwise linear regression coefficients
**Hooberman et al.,**[34]	75 torture-victim refugees in US from Asia (15), Africa (53), Europe (6) and South America (1)	MSPSS, SES, Cognitive appraisal measure. Moderator variable of coping measured via CSI-SF	HTQ for PTSD	Translated/back translated in to French/Tibetan, interpreters used. HTQ Cα;0.88	Hierarchical regression models to test moderator effect on resilience variables.
**Andersson,**[26]	98 Finnish adult evacuees from World War II and 54 non-evacuees	EMBU	PCL-C	Swedish versions used. PCL-C Cα;0.90, EMBU Cα; 0.90	t-test (2 tailed), *X*^*2*^ test, Pearson's correlation coefficient, OR
**Araya et al.,**[35]	749 displaced women in Addis Ababa and 110 displaced women in Debre Zeit, Ethiopia.	Perceived social support captured through Social Provisions Scale, Coping strategies, WHOQOL-BREF	SCL-90-R, WHOQoL-BREF, HTQ section 1	Translated to Amharic and culturally validated.	t-test, *X*^*2*^ test, Multivariate binary logistic regression coefficients
**Beiser et al.,**[36]	1603 Sri Lankan Tamils in Toronto, Canada	Pre/post migration stressors, family-based social support, perceived quality of life	WHO-CIDI-K for PTSD	Translated and back translated to Tamil. Interviews conducted in English/Tamil on preference.	Descriptive statistics, OR and AOR
**Bhui et al.,**[28]	142 Somali refugees in London, UK	CPQ	MINI, Discrimination experiences, Residential mobility	Translated and back translated to Somali with further tests for reliability/validity	Descriptive statistics, OR
**Suarez,**[37]	151 Quechua women from Ayacucho, Peru including displaced, non-displaced and returnees	CD-RISC	HTQ-GEV & HTQ-PTSD-R for PTSD symptoms, TQ-LID, LSQ, socio-demographics	Translated and back translated from English to Spanish to Quechua. Assessed for cultural and semantic validity, used previously validated HTQ and TQ-LID	Descriptive statistics, Hierarchical regression
**Arnetz et al.,**[27]	75 Iraqi refugees and 53 non-Iraqi Arab immigrants in Michigan, US	RS 8-item version	Modified GHQ, PCL for PTSD, Exposure to violence,	Translated and back translated to Arabic. Questionnaire completed in Arabic by participants	*X*^*2*^ test, t-test, Mann–Whitney U-test, Linear regression

*SOC-13 - Sense of Coherence 13 item version; HSCL-25 - Hopkins Symptom Checklist 25 item version; PDS - Bilingual PTSD and Posttraumatic Stress Diagnostic Scales; GHQ-12 - General Health Questionnaire 12 item version; TQ - Trauma Questionnaire; RS-11 - Resilience Scale 11 item version; RS-8 - Resilience Scale 8 item version; PHQ-2 - Patient Health Questionnaire Depression Module; GAD-7- Generalised Anxiety Disorder; FLZ^M^ - Questions on Life Satisfaction German version; WHO CIDI-K - WHO Composite International Diagnostic Interview Section K for PTSD; MSPSS - Multidimensional Scale of Perceived Social Support; SES - Self-Evaluation Scale; CSI-SF - Coping Strategies Inventory-Short Form; HTQ - Harvard Trauma Questionnaire; EMBU - Swedish acronym for “Own Memories of Parental Rearing”; PCL-C - Post Traumatic Stress Disorder Checklist-Civilian version; SCL-90-R - Revised Symptom Check List; WHOQoL-BREF World Health Organization Quality of Life; CPQ -Close Persons Questionnaire; MINI - Mini Neuropsychiatric Interview; CD-RISC - Connor-Davidson Resilience Scale; LSQ- Life Stress Questionnaire.

**Cα - Cronbach's Alpha; t-test - Student t-test; RR - Risk Ratio; *X*^*2*^ test - Chi Square test; MANOVA - Multi Variate Analysis of Variance; OR - Odds Ratio; AOR - Adjusted Odds Ratio.

**Table 3:** Summary of quantitative study findings

**Study**	**Resilience outcomes**	**Mental health outcomes**	**Conclusion**
**Almedom et al.,**[29]	Considering SOC-13 results, resilience is low among those who live in IDP camps, and significantly low among women (more so for women living in IDP camps).	Although no specific mental health outcomes were explored, findings show critical implications for health policy covering prolonged forced displacement.	Highlights the need for international health institutions including the WHO and local players to address the plight of IDP women, particularly in conflict and post-conflict zones.
**Jamil et al.,**[31]	Resilience is discussed in the light of two case studies presented along with quantitative analyses for mental disorder symptoms. Pre-migration and post-resettlement stressors have a strong impact on resilient behaviours.	Many refugees met criteria for the diagnosis of PTSD (54.5% of the men; 11.4% of the women). 34.3% of women and 4.3% of the men were diagnosed with a depressive disorder. The HSCL-25 showed more than 80% of participants had recently experienced intense symptoms of anxiety.	Primary medical care service providers need more education and training to screen refugees for mental health services. Important to have culturally-sensitive screening and diagnostic instruments.
**Almedom et al.,**[30]	Using the SOC-13 to measure resilience quantitatively, findings show that urban (non-displaced) residents and rural, traditionally mobile (pastoralist) communities had significantly higher resilience than those living in IDP camps. Findings show that displacement can compromise individual or collective resilience among women.	No specific mental health outcomes were explored. However, findings points to the fact that displacement is detrimental to the mental well-being of conflict survivors of war. Especially, the prolonged duration of the internal displacement in Eritrea (5–6 years), has been damaging .	Displacement may compromise individual and/or collective resilience in women. Health research should contribute to the promotion of resilience factors in post-conflict countries as part of public health policy.
**Pedersen et al.,**[25]	Using a mixed-method approach, protective influences derived from resilient structures in societies involved in survival and conflict resolution is explored.	High levels of mental disorders (anxiety, depression, PTSD) were identified. Significant associations were observed between degree of exposure to violence and the likelihood of developing mental illness. Negative association between degree of social support and mental health outcomes was also observed.	Highlights the need to look beyond PTSD and focus on culture-specific trauma-related disorders and long-term effects. Discusses the need for further research to establish social bonds, strengthen support networks and increase social cohesion in societies damaged by trauma and dislocation.
**Kuwert et al.,**[32]	Using the RS-11, study shows that displaced individuals have significantly less resilience levels than their non-displaced peers.	Even sixty years after WWII, displaced individuals showed significantly more anxiety symptoms than the non-displaced population. Displaced participants also had higher levels of depressive symptoms, albeit statistically not-significant.	Study highlights the long-lasting impact of forced displacement on mental health in the now elderly German population. Provides strong evidence on the need for preventive measures and effective interventions for elderly forced migrants.
**Beiser et al.,**[33]	The study included measures of social capital as elements of community resilience. Perceived social support is shown to reduce the probability of PTSD, along with feelings of safety and perceptions of moral and social order. Persistence of PTSD was partially attributable to the loss of social capital due to conflict-induced disintegration of social fabric.	The six-month period prevalence of PTSD in the violence-affected village was 60%, more than four times higher than the non-affected village. A dose–response relationship is evident between exposure to human-induced conflict/disaster and mental health.	Conflict-induced social and cultural disintegration can lead to lowering of community resilience, and continuing mental health issues.
**Hooberman et al.,**[34]	Results indicate that relevance of resilience variables can depend on individual coping style. Emotion-based coping styles showed moderating effects between PTSD and cognitive appraisal, social comparison variables.	40% of the sample showed above cut-off scores on HTQ for PTSD.	Cultural variations and overlap between PTSD symptoms and coping modes limits wider interpretations. However, clinical implications point towards using coping styles and cognition in managing PTSD among refugees surviving torture.
**Andersson,**[26]	Resilience is not directly measured. EMBU and its outcomes on parental separation and rejection is used as a proxy measure of resilience process. Indicates the need for more exploration of childhood detachment experiences among traumatized populations and the link to the process of resilience.	65 years after the end of WWII, the Finnish refugees had a 10 times higher risk for PTSD when compared to non-evacuees. A significant proportion (36.7%) refugees had experienced extreme traumatisation.	Resilience process and the link to childhood parental separation and extreme trauma require further in-depth attention.
**Araya et al.,**[35]	The process of resilience is seen to be positively influenced by the placement of displaced persons in a community setting Task-oriented coping, higher perceived social support, and a favourable marital life associated with a markedly higher quality of life promote the resilience process.	Mental distress, assessed by SCL-90-R, did not significantly differ between the two groups.	Findings suggest that community setting-based living and rehabilitation improves quality of life for post-conflict displaced populations. Improvement in living conditions may also improve quality of life in camp-like shelters.
**Beiser et al.,**[36]	Family-based and non-family based social support together with perceived quality of life was used to explore resilience outcomes. Life satisfaction and non-kin support was associated with resiliency and demonstrated a reduction in PTSD prevalence.	ICD-10 criteria based lifetime prevalence for PTSD was 12%; DSM-IV criteria based lifetime prevalence was 5.8%. Pre and post migration stresses increased the risk of PTSD.	Study underlines the importance of understanding resilience and its sources, most notably social support, in relation to developing PTSD.
**Bhui et al.,**[28]	Using social support networks as an indicator of resilience, study provides evidence that larger (stronger) support networks promote resilience against developing mental disorders, especially salient in situations of high forced residential mobility for refugees.	Significant associations evident for any mobility with general health, trauma history and any psychiatric diagnosis. Forced residential mobility more likely to be associated with ICD-10 criteria based psychiatric disorder compared to self-choice mobility.	Social support networks may promote resilience among refugees experienced forced residential mobility and associated mental disorders.
**Suarez,**[37]	Resilience contributed to the variance of avoidance symptoms but not to the variance of PTSD symptoms, re-experiencing or arousal. The CD-RISC mean scores in the sample were lower than that of a national community sample in the US.	Only 9.3% showed possible PTSD with scores above the 2.5 HTQ cut-off. LSQ score showed a moderately high level of life stress among the participants.	Complexity of interactions between resilience and post-traumatic responses are shown. The resilience shown by the women in the study calls for more recognition of women's roles in post-conflict societies.
**Arnetz et al.,**[27]	No differences were seen in resilience between Iraqi refugees and non-Iraqi immigrants. Resilience was a important inverse predictor of psychological distress when controlled for migration and exposure to violence, but not for PTSD.	Refugees had shown more PTSD symptoms compared to immigrants.	Resilience and its association with decreased psychological stress is important in managing victims of conflict.

**Table 4:** Overview and summary of qualitative study findings

**Study**	**Sample and background**	**Resilience findings**	**Mental health findings**	**Conclusion**
**Davis et al.,**[38]	19 adult Southeast Asian (Vietnamese, Cambodian and Laotian) women in central Pennsylvania, US	Pre and post migration experiences were explored. Cultural bereavement, post-migration adversity, despair and isolation were overcome with different survival strategies. Family cohesion and adaptation highlighted as promoters of resilience.	None of the participants were seen to be suffering from PTSD related to traumatic displacement. Study argues that it is largely the lack of same ethnic communities and family support systems that may lead to the development of mental health issues.	Recognition of cultural bereavement by health workers and development of interventions that involve ethnic and cultural identity is important to promote resilience and mental well-being.
**Schweitzer et al.,**[39]	13 resettled Sudanese refugees in Australia, aged 17-44	Several strengths and resources that allowed coping with migration stressors for refugees were identified: family and community support; religion; personal qualities, and comparison with others. These can act as promoters of resilience against the development of psychological sequelae of forced displacement.	Forced displacement creates significant psychological stressors during pre-migration, transition and post-migration periods.	Coping strategies form an important part of resilience in response to trauma and forced migration experience. Identifying these factors are important in formulating strategies to improve the well-being of resettled refugees. However, small sample size and heterogeneous sample limits interpretation.
**Sossou et al.,**[40]	7 Bosnian refugee women resettled in Southern US	Narrative analysis identified several resilience factors: importance of family and values, role of spirituality as a strength through non-organized religion and community support services during resettlement.	The study aimed to explore general wellbeing in the backdrop of prior trauma. Life and experiences during war, challenges during resettlement such as misconceptions on mental health services were indicated as potential reasons for poor mental health.	Life experiences during and post-war and resettlement experiences may lead to poor mental health. Family, spirituality and social support can be resilience promoting factors for these female refugees. However, the small sample size limits wider interpretation.
**Somasundaram & Sivayokan,**[41]	IDPs in Vanni, Sri Lanka	An exploration of collective trauma experienced during forced displacement and conflict. Resilience and post-traumatic growth develops in spite of severe traumatic experience of displacement and resulting breakdown of family/community network and structures.	Severity of the forced displacement episode leads to the development of psychosocial symptoms including PTSD.	Interventions for psychosocial regeneration are required to rebuild the family and community structures in the aftermath of mass displacement including the healing of memories.
**Nuwayhid et al.,**[42]	IDPs in Lebanon	Community resilience explored by combining direct observation, key informant discussions and review of material. Community resilience is suggested as a process rather than an outcome. Resilience is built upon collective identity, previous war experience and social support networks.	Links between resilience (community or individual) were not explored. However, the impact of sudden forced migration on psychosocial health of communities is noted.	Implications for public health professionals to build community resilience is discussed. Capitalising on community resilience a key component of public health action.
**Thomas et al.,**[43]	16 Pakistani and 8 Somali urban refugees in Nepal	Primary relationships along with supportive networks of friends and family members facilitated coping mechanisms, functioning as a mode of resilience for many. These provided a buffer against vulnerabilities and reduced anxiety through psychological support. Religion also played a similar role in promoting resiliency.	Psychosocial distress of being a refugee was explored. Vulnerability was characterised by discrimination, daily stressors, unfulfilled expectations, and lack of control, culminating in generally poor reported mental health.	Culturally relevant programmes that seek to develop esteem and build resilience should be developed alongside individualised therapy for those who are vulnerable. External support should be designed in a way that builds resilience and facilitates coping.
**Fernando,**[44]	43 Sri Lankans	Resilience construct examined through focus groups. Some elements of resilience are common across ethnic-cultural groups while other differed across ethnicities. Two distinct non-western resilience components identified were psychosocial gratitude and strong will linked to religion or karma. Certain resilience components can be taught.	Links between type of trauma and components of resilience identified.	Components of resilience, and understanding of resilience can vary across ethno-cultural groups. Public health interventions and policies can make use of components of resilience that can be taught to populations experiencing trauma.
**Lenette et al.,**[45]	4 single African (Sudan, Burundi, Democratic Republic of Congo) refugee women in Australia	Resilience ethnographically explored as a social process linked to every-day life in the context of interactions between individuals and environment. Resilience is identified as an inter-subjective process connecting refugee women with their environment through social spaces. Nature and dynamicity of resilience is described. Social complexities in resilience and stress is discussed.	Mental health is not specifically explored or studied. However, pre-migration stressors and post-migration stressors such as daily living, coping, and resettlement are discussed.	The findings argue for more attention to resilience pathways and outcomes linked to day-to-day lives of refugees, which can be useful in developing refugee mental health practices.
**Chung et al.,**[46]	9 single, low-income refugee women (Hungary, Nigeria, Iraq, Cameroon, Afghanistan, Sudan, DR Congo) in Ontario, Canada	Study explored how resilience is grown, promoted or can be reinforced. Through a grounded theory approach, findings show that informal, formal support and individual characteristics of refugee women reinforce resilience. Findings support a collective resilience model.	No specific mental health issues were explored. Links were made with migratory and post-migratory stresses.	Organizational and social support reinforces resilience. Individual characteristics are an important factor in sustaining resilience. Collective resilience require further exploration.
**Lewis,**[47]	80 Tibetan exiles living in Dharamsala, India	An ethnographic study exploring resilience among Tibetans in exile, a community known to be highly resilient to trauma. Tibetans consider resilience as an active and learned process, and use Buddhist thinking to exempt negative influence of trauma.	Traumatic experiences instigated by torture, violence and displacement were explored.	Findings challenge the idea that trauma is inevitable in conflict or political violence and that some communities dispel or transform distress.

The ten qualitative studies used convenience sampling methodology [38-47]. The 23 studies were conducted in varied geographical locations: Australia (Sudanese, Burundian and Congolese refugees), Canada (Sri Lankan Tamil, Hungarian, Nigerian, Iraqi, Cameroonian, Afghan, Sudanese and Congolese refugees), Eritrea, Ethiopia, Finland, Germany, India (Tibetan refugees), Lebanon, Nepal (Pakistani and Somali refugees), Nigeria, Peru, Sri Lanka, United Kingdom (Somali refugees), United States of America (Asian, African, European and South American refugees). Three studies were conducted on internally displaced [35,41,42] and 11 were conducted among refugee/asylum seeker populations [28,31,34,36,38-40,43,45-47]. Nine mixed-population studies were included [25-27,29,30,32,33,37,44].

Resilience was measured in quantitative studies using the following instruments: the Close Persons Questionnaire (CPQ) [28], self-designed questionnaires [33,35,36], the EMBU – a Swedish acronym for “Own Memories of Parental Rearing” [26], an adapted Sense of Coherence Scale (SOC-13) [26,27], two versions of the Resilience Scale (RS-11, RS-8) [27,32], the Connor-Davidson Resilience Scale (CD-RISC) [37] and with variables from Multidimensional Perceived Social Support Scale (MSPSS), Self-Evaluation Scale (SES) and Coping Strategies Inventory-Short Form (CSI-SF) [34].

Mental health outcomes were measured using the following instruments: the Mini Neuropsychiatric Interview (MINI) [28], the WHO Composite International Diagnostic Interview Section K for PTSD (WHO-CIDI-K) [33,36], the Revised Symptom Check List (SCL-90-R) [35], the Post Traumatic Stress Disorder Checklist-Civilian version (PCL-C) [27,35], the 25 item Hopkins Symptom Checklist (HSCL-25), the Bilingual PTSD and Posttraumatic Stress Diagnostic Scales (PDS) [25,31], the Patient Health Questionnaire Depression Module (PHQ-2) [32], Generalised Anxiety Disorder (GAD-7) [32], General Health Questionnaire-12 (GHQ-12) [25,27], Trauma Questionnaire (TQ) [25,37], Harvard Trauma Questionnaire [34,37] and Life Stress Questionnaire [37].

The review findings are categorised and presented separately by quantitative and qualitative study designs. The single mixed-method study is described under both these headings, separated by its qualitative and quantitative components respectively.

### Qualitative studies exploring resilience and mental health

The ten studies identified in the review included refugee [38-40,43,45-47], IDP [41,42] and mixed-population studies [44]. Five studies were conducted in HIC settings [38-40,45,46] and the other five in LMIC settings [41-44,47]. There was a wide variation in sample sizes, which ranged from 4 to 80. The qualitative component of the sole mixed-method study was conducted in a low-income setting using in-depth interviews aided by ethnographic methods and had a sample size of 45 participants [25].

The qualitative studies explored resilience and mental health mainly via individual interviews. However, some studies used focus groups, informant discussions and direct observations, using additional complimentary data sources where appropriate. Ethnographic methods were used by some studies [45,47]. Findings and conclusions from these studies in relation to resilience and mental health are summarised in Table 4. Resilience in these populations affected by conflict-driven forced migration was concluded to be influenced by social complexities, family and community cohesion, family and community support, individual personal qualities, collective identity, supportive primary relationships and religion. Psychosocial stress caused by the often traumatic displacement ordeal was described as having the most detrimental effect on mental health. However, individual and community resilience in these populations were found to have acted as a deterrent, protecting the majority of forced migrants against poor mental health outcomes. Elements of resilience was shown to be common across ethno-cultural divides while some components of resilience could be taught [44]. Cultural and gender-specific differences and the role of everyday-stress in resilience processes and dynamics were identified [45-47].

### Quantitative studies exploring resilience and mental health

A summary of resilience and mental health outcomes is presented in Table 3. Five studies used direct individual resilience measuring instruments [27,29,30,32,37]. Community resilience was assessed in two studies [33,41]. Two studies explored resilience outcomes among exclusively-female forced migrant populations [35,37]. An association of low resilience with female gender was reported by two studies [29,30]. Living in IDP camps was associated with low resilience in three studies [29,30,35]. Social support, capital and networks were used as indirect measures of resilience in several studies [28,33-36]. One study looked at parental separation and rejection in conflict-evacuees and subsequent link to resilience process [26].

Three studies included in the review looked at direct associations between resilience-related factors/variables and mental health outcomes [27,34,37] and two of them used resilience-specific measurements [27,37]. Most studies found relatively high levels of mental disorders among the population concerned and focused on a PTSD diagnosis [25-27,31,33,34,36,37]. The most commonly studied disorders were PTSD, depression and anxiety. Several studies focussed only on pre/post migration stressors or psychological distress, instead of specific mental disorders [29,30,35]. One study looked at forced residential mobility of a refugee population and how psychological and general health is affected [28]. Pedersen et al., [25] explored the degrees of exposure to violence and the link to mental health albeit without adjusting for the potential mediating role of resilience.

Sample sizes of selected studies varied widely from 75 to 1603, although most studies provided appropriate sample size calculations. Convenience sampling was used in two studies [26,37], while others used randomly selected samples. Response rates were high in most studies, with 100% participation reported in one [33]. Reliability and validity information on measures used in the studies were largely limited to the provision of Cronbach alpha scores, although some studies described cultural and other forms of validation of instruments prior to data gathering while some studies had used bilingual interpreters (see Table 1) [25,28,29,33-35,37].

## Discussion

This study systematically reviewed the current global literature on resilience and mental health in adult displaced populations. Studies identified by the search varied widely with regard to samples, context, study design, measurements, approach to data analysis, and whether the primary study focus was on resilience or mental health of displaced persons. Therefore a narrative synthesis approach was adopted to capture this heterogeneity.

The critical nature of resilience - encompassing perceived and available social support, sense of coherence, social networks, coping strategies, individual qualities, religious belief systems and culture - and its collective positive impact on the psychosocial health of the displaced adults was highlighted in reviewed findings. High quality social support and family support was shown to be associated with increased resilience and lower levels of psychological problems in all phases of conflict-induced forced migration. Evidence also points towards factors associated with weaker or stronger resilience, especially the roles of gender and daily stressors. Prolonged displacement is shown to have a negative impact on levels of resilience, adding to the impact of the camp-like post-displacement living conditions. This reflects findings on the effect of forced displacement on mental disorders [3]. Another critical finding to emerge is the evidence on how parental separation or rejection at times of conflict/forced migration can affect levels of resilience and psychosocial well-being in later life. More importantly, evidence has been generated on community resilience and its impact on the collective negotiation of traumatic experiences by displaced communities, effectively reducing the overall burden of mental illnesses [41,42]. In fact, the study of collective resilience through qualitative methodology bears vital implications in understanding the diversity of the resilience construct among populations experiencing adversity, and the development of resilience-promoting interventions [48,49].

Through the emerging evidence from the review, a theoretical framework for resilience-mental health interaction can be conceptualized. It represents key factors negatively affecting both resilience and mental health of displaced populations and also captures key supportive factors identified from reviewed studies. Factors that influence low resilience or poor mental health are termed 'undermining' and include levels of acculturation, daily stressors, breakdown of family, social & cultural networks, living conditions (e.g. IDP), gender and continuing displaced status. Those that influence higher resilience and positive mental health are termed 'supportive' and include sense of coherence, higher family and social support, strong family and social networks, coping (individual/communal), religion and belief systems, individual (personal) qualities and strengths and community support. The interdependent, dynamic association between resilience and mental health, as shown through the review findings, are parts of the this framework.

The emerging theoretical framework highlights the social-ecological construct of resilience that argues for contextualising resilience as a product of supportive environments with sufficient resources that aid individuals (or communities) to overcome adversity [17,50]. In addition, it highlights the dynamic, multilevel, multi-contextual nature of resilience that follows and develops along individual and communal trajectories of adversity [17,23,50,51]. However, causal pathways and directionality cannot be accurately represented due to insufficient evidence found in the review.

This review has identified some gaps in the evidence base of this field, where further research may be beneficial. Adult resilience, especially resilience trajectories across the life-span of those who experience forced migration appears to have received less attention, limiting the potential understanding of resilience dynamics in the long-term. Only a few studies have used resilience-specific measurements, opting instead to use social support and other proxies. This may reflect the lack of a clear definition of the resilience construct, confusion about its core components and the lack of specific tools [52]. A methodological review of resilience measurement scales indicated that there is no 'current gold standard' among those reviewed, and a number of them require further development and validation, which may be indicative to researchers not using resilience-specific instruments [53]. In addition, none of the studies sought to define or conceptualise resilience through their findings, which is an important limitation given the amorphous nature of the construct [54]. More importantly, the usage of existing measurements in cross-cultural settings has been minimal, effectively limiting the scope of their usefulness, an important issue given the hugely diverse nature of cultures and countries affected by conflict-driven forced migration over the years [11,53,54]. Most reviewed studies using indirect measures of resilience tended to extrapolate their findings to the broader concept of resilience, risking adding more confusion to the existing definitional debates [52,55].

Another gap identified in the review is the lack of longitudinal and interventional studies on resilience of adult forced migrants. The lack of longitudinal data is problematic for several reasons: i) it prevents the effective understanding of resilience dynamics over time, especially within the post-displacement period where migration-related and daily living stressors may have a defining impact on the process of resilience; ii) it limits the identification of the temporal nature of protective or promoting factors of resilience, reducing its dynamic nature to a static concept [56,57]; iii) it limits conclusions regarding causality; iv) it hampers the development of effective, evidence-based interventions aimed at promoting resilience among vulnerable groups of forced migrants, especially the elderly [57]. However, the difficulties and ethical dilemmas in conducting longitudinal studies in highly unstable and chaotic conflict or post-conflict situations has to be acknowledged in this regard.

This review identifies the need to recognise and enhance the resilience of displaced persons as a priority for intervention developments, particularly in resource-poor settings. For example, educational intervention programmes to promote the resilience and psychosocial health of conflict-displaced youth have been recognised as effective [23,58]. This model of resilience can be applicable and used on a global scale, even among adult populations with necessary adjustments. However, such interventions require effectiveness measuring through randomised controlled trials before full implementation. A key implication for any rehabilitation programme of forced migrants is the preservation and promotion of resilience, whether via the restoration of social bonds, strengthening support networks or through increasing social cohesion in families, communities or societies displaced by conflict.

Although the current review confirmed earlier findings that displacement can have an adverse impact on the mental health of displaced populations when compared with non-displaced groups [3,8], only two studies had actually linked adverse mental health outcomes with resilience of those affected [27,37]. The mental health focus of studies has been largely on PTSD, anxiety and depression. The need to explore a wider range of mental disorders is highlighted by the review findings, as the concept of PTSD incompletely capture the psychosocial experiences of forced migrants [11,54,55,59]. Effort is needed to conduct studies that explore more intricate associations between resilience and mental health, especially positive mental health [23,60]. The focus on a medicalised model of trauma and recovery may be an obstacle to a adopting a more holistic approach towards forced displacement and resulting mental health issues that includes resilience promotion [55,61,62]. Western concepts of responding to trauma are not necessarily applicable to local, indigenous and culturally-specific methods of resiliency, coping and survival strategies which should not be disregarded [25,47,63-65]. We argue that the concepts and measures of resilience, and related interventions, should be locally derived [47]. However, researchers should be equally wary about conducting interventions focused solely on resilience, without linking them treatment interventions, particularly in more extreme situations [66].

This review identifies several other deficiencies in current knowledge. More attention should be given to presenting gender and age disaggregated quantitative data, given the differences in mental health outcomes between genders and the possibility of similar differences in resilience, as well as due to the fact that worse mental health outcomes are reported for older conflict-affected populations and potential similarities in resilience-related outcomes. In addition, studies need to look at resilience in low income settings in comparison to high income settings, while different conflict-affected populations such as refugees and IDP living in camp-like, urban and other settings need further exploration. Although our review includes studies conducted in both income settings, the evidence available is insufficient to draw any firm conclusions about differences in resilience between them, due to variations in study design, sample sizes and variables used.

This review found only two studies exploring community resilience [41,42] while majority focused on individual resilience, highlighting the need to explore this concept according to its type (e.g. family support; social support; supportive environment; promotive vs. protective resilience). One study included in the review explored correlation between individual and collective identity, and its link to national level resilience [42]. This study provides evidence of how, individual, collective and national resilience can have a positive impact on mental health at populations levels, with important public health implications [42]. The social and ecological aspects of resilience can be useful in developing national or country level responses to adversity, increasing the possibility of positive overall mental health outcomes for affected populations [14].

The findings show a marked lack of mixed-methods studies, given the value of combining quantitative and qualitative study methods in resilience research [19,54,67]. Quantitative studies with increased sample sizes allowing for in-depth analyses along with more intervention studies are encouraged [19]. While resilience is commonly referred to, including in the leading guidelines (IASC) in the literature exploring conflict-affected populations, this review shows there is paucity of evidence (including tools and definitions) on the nature and role of resilience in such settings and the relationship with mental health outcomes [67].

### Limitations in the review

The diverse study designs and methodology across the reviewed publications restricted the cross-applicability of findings and makes definitive generalisations difficult, and the diversity of resilience measures and outcomes precluded meta-analysis for the quantitative studies. The number of studies selected for the final in depth review was not extensive enough to provide conclusive evidence about the pathways of resilience impacting on the mental health of forced migrants. We adopted a realist review approach to counter the diverse nature of selected studies and to maximise the utility of findings in the light of the complexity of the review topic [68]. The narrative synthesis was seen as the most appropriate in the study context. There is a risk of missing important studies published in languages other than English, which is a limitation for its generalisability. The search terms selected and used in the review may also have limited the number of positive results, although we tried several different combinations to avoid this happening. A quality review was not conducted, due to the wide variation of studies found. The review also did not include outcomes related to functioning and it would be useful for further studies to explore overall functioning, rather than focus only on mental disorders and symptoms in conflict-affected populations.

To conclude, the complex processes of resilience among displaced adults in the presence of substantial adversity – and their impact on mental health – remain unclear in the literature and require further research. This review provides evidence on the positive role of resilience on the specific and general mental health of conflict-driven displaced populations in the global context. The review identifies the need to enhance resilience of adult displaced populations as a priority for intervention development, particularly in resource-poor settings. The pathways of resilience and dynamics of resilience among forced migrants in conflict settings need to be further elucidated and researched as a priority for intervention programmes. The construct of resilience require further exploration with regard to displaced populations, especially affected by prolonged displacement. Methodological and theoretical flaws require addressing, and researchers, humanitarian workers and other stake holders would benefit from a further examination of the resilience construct and its link to mental health in displaced populations.

## Competing interests

The authors declare that they have no competing interest.

## Authors’ contributions

CS conceptualized the review. SSA performed the search and wrote the first draft. CS reviewed and wrote the second draft. BR & RS reviewed and edited the second draft. All authors reviewed and approved the final manuscript version.
